# Unusual oral manifestation of Kindler syndrome: a case report and review of literature

**DOI:** 10.3389/froh.2024.1430698

**Published:** 2024-09-05

**Authors:** Rahul Bhandary, Geethu Venugopalan, Padmaraj Hegde

**Affiliations:** ^1^ Department of Periodontology, A B Shetty Memorial Institute of Dental Sciences, Nitte (Deemed to be University), Deralakatte, India; ^2^ Department of Oral and Maxillofacial Surgery, A B Shetty Memorial Institute of Dental Sciences, Nitte (Deemed to be University), Deralakatte, India

**Keywords:** Kindler syndrome, pyogenic granuloma, periodontitis, oral mucosal diseases, integrin dysfunction

## Abstract

Kindler syndrome (KS) is a rare autosomal recessive genodermatosis characterized by congenital acral blistering, that typically presents in infancy and is followed by the development of characteristic poikilodermatous pigmentation and photosensitivity in later life. These clinical manifestations arise from mutations in the FERMT-1 (Fermitin family homologue 1) that encodes kindlin-1, a protein localized to focal adhesions in keratinocytes. Kindlin-1 plays a crucial role in integrin receptor activation, which is essential for cell adhesion and migration. Most KS cases exhibit reduced or absent kindlin-1 expression, leading to defective integrin activation and impaired cell adhesion and migration processes. This impaired cell adhesion ultimately results in the blistering phenotype observed in KS. Oral manifestations of KS are frequently under-reported and misdiagnosed, potentially leading to delayed or incorrect treatment. Furthermore, diabetes mellitus (DM) can further exacerbate the severity of KS due to impaired epidermal barrier function and compromised periodontal health. This co-morbidity creates a synergistic effect. Periodontal infection, often exacerbated by DM through a caspase-3-dependent mechanism, can cause apoptosis of epithelial cells and fibroblasts. This enhanced apoptosis and loss of epithelial barrier function due to DM further hinder tissue repair processes. Consequently, both cutaneous and oral complications associated with KS become more severe in diabetic patients. We report a unique case of a diabetic adolescent with KS presenting with a massive oral pyogenic granuloma and extensive periodontal destruction with a comprehensive review of the literature exploring the current understanding of oral manifestations in KS, emphasizing their under-diagnosis and potential for exacerbation by DM. This case emphasizes the need for increased awareness of oral manifestations in KS, especially in diabetic patients. Early diagnosis and a multidisciplinary approach are crucial for optimal management of cutaneous and oral complications associated with KS.

## 1 Introduction

Poikiloderma of Kindler, also known as Kindler epidermolysis bullosa (ORPHA:2908; ICD-11: LD2B; OMIM: 173650; UMLS: C0406557; MeSH: C536321) is a rare inheritable genodermatotic disorder, and the fourth major subtype of Epidermolysis Bullosa. KS arises from recessive loss-of-function mutations in the FERMT1 gene (1). This gene encodes kindlin-1, a protein crucial for integrin activation and subsequent cell adhesion and migration processes within keratinocytes. Mutations in KIND1 lead to reduced or absent kindlin-1 expression, ultimately disrupting these essential cellular functions. Affected individuals typically present with a constellation of cutaneous manifestations including skin atrophy, progressive poikiloderma (characterized by atrophy, telangiectases, and reticular pigmentation), and photosensitivity with erythema and photo-induced blisters in early childhood, often diminishing after adolescence (2).

The deficiency of the Kindlin-1 in basal keratinocytes of oral epithelia leads to diminished integrin expression in the junctional epithelium, which is critical for facilitating cell migration and adhesion via focal contact at the tip of the Directly Attached to Tooth (DAT) cell (3). This impaired gingival junctional epithelium adhesion to the tooth surface (via DAT cell) predisposes the individual to chronic gingivitis and periodontitis (4, 5).

This case report describes a patient with Kindler syndrome (KS) and Type 1 diabetes mellitus (DM), highlighting the severe oral manifestations potentially arising from the synergy between these conditions. We emphasize the need for clinicians to consider potential systemic implications of dermatologic and other systemic manifestations such as DM when formulating oral treatment plans, as solely addressing oral conditions may be insufficient for optimal long-term management.

We also aim to highlight the potential consequences of neglecting recall appointments and inadequate parental reinforcement of oral hygiene practices. Timely intervention with scaling and root planing (SRP), coupled with consistent home oral hygiene practices, could have improved the oral health of the patient. However, the patient presented for treatment only at a stage of severe bleeding and pain, indicating a lack of awareness or neglect about the importance of regular preventive dental care, thus highlighting the critical importance of comprehensive patient/caretaker education. It emphasizes the necessity for dental professionals to prioritize patient education, particularly for parents or caregivers, to ensure proper oral hygiene practices and regular dental visits. Early intervention through education, counseling, and motivation of parents/caregivers regarding the importance of dental care could significantly improve the long-term periodontal prognosis, and enhance the overall oral health-related quality of life.

## 2 Case report

A 14-year-old adolescent female of South Asian descent presented to us following referral from a local hospital, with a chief complaint of a gradually progressing swelling in the maxillary anterior region associated with persistent bleeding and intense discomfort following a prophylactic ultrasonic scaling procedure.

The patient had a strong family history of type 1 diabetes, with both the mother and maternal grandmother having the condition. In addition, the parents and grandparents were all first cousins, indicating a long lineage of consanguinity. She weighed about 20 kilograms and had a BMI of 10.2, putting her BMI for age below the first percentile.

Initial laboratory examinations revealed hyperglycemia, ketonuria, glycosuria, hyponatremia, Hypovitaminosis D, mild microcytic hypochromic anemia with neutrophil leucocytosis, myeloid left shift, and thrombocytopenia (Table 1).

**Table 1:** Data from laboratory investigations.

Test	Result	Reference values
Haemoglobin	10.4	11.6 to 15
Leukocyte total count	13.2	4.5–11.0 × 109/L
MCHC	31.7 g/dl	34 ± 2 g/dl
MCH	23.9 picograms/cell	27–31 picograms/cell
MCV	75.4 fl	80–100 fl
Packed Cell Volume	33.00%	35.5 to 44.9%
Platelet Count	5,15,000	150,000 to 400,000/ml
RDW	16.9%	12%–15%
RBC count	4.37 million/cumm	3.8 to 5.2 million/cumm
Neutrophils	74%	55% to 70%
Lymphocytes	17%	20% to 40%
Eosinophils	3%	1%–4%
Monocytes	6%	2%–8%
Basophils	0	0–0.5%
Serum Urea	37	18–45 mg/dl
Serum creatinine	0.69	0.45 to 0.81 mg/dl
Serum sodium	125	35 to 145 (mEq/L)
Serum potassium	3.41	3 and 3.5 mEq/L
Serum chloride	82.6	90–110 mEq/L
Serum bicarbonate	21.5 mEq/L	21–26 mEq/L
Plasma Glucose Random	524 mg/dl	200–250 mg/dl
HbA1c	7.74%	7.5%

**Figure 1:**
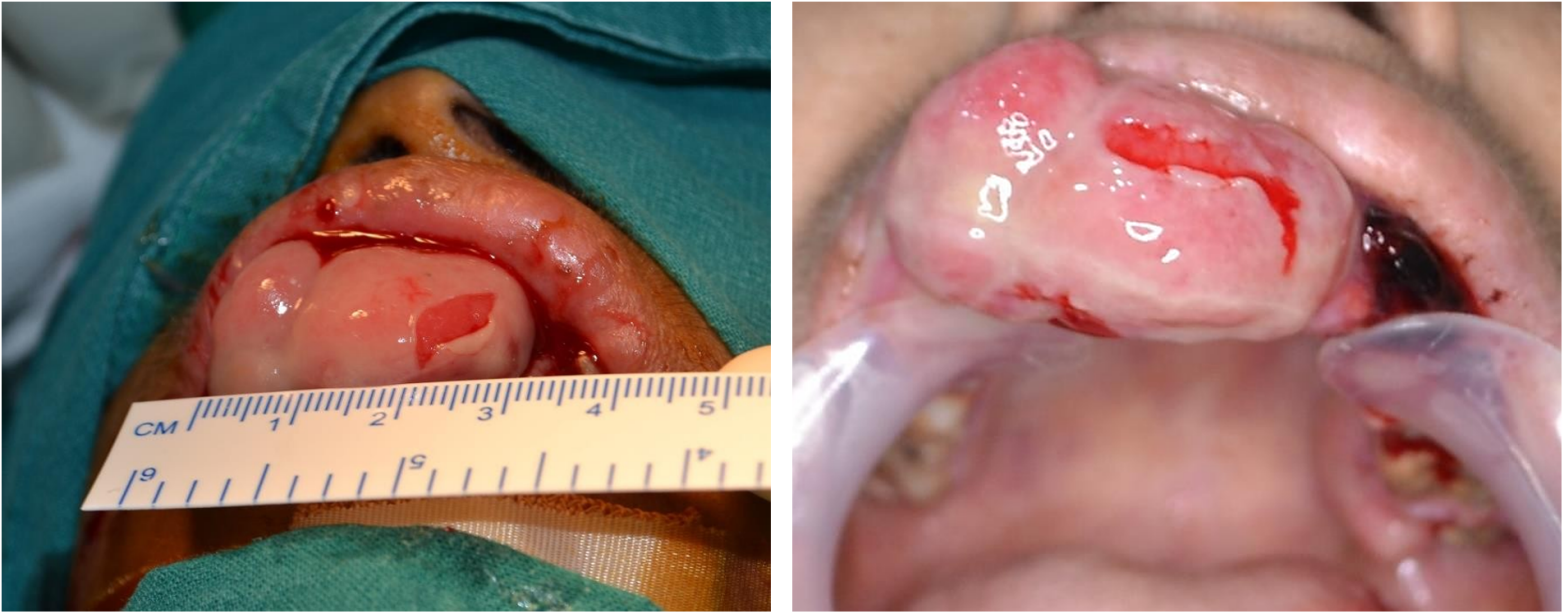
Massive pyogenic granuloma in the maxillary anterior region. The image depicting the massive pyogenic granuloma of approximate size 21 × 34 cm (Length × Width) in the maxillary anterior region shows fragile eroded mucosal lining areas and profuse bleeding.

On intraoral examination, a pinkish-red sessile, lobulated, and erythematous enlargement measuring approximately 21 mm × 34 mm was observed in the maxillary anterior region, encompassing both the vestibular and palatal sides, covering the crowns of the affected teeth. Several ulcers were also noted in the vestibular region. The enlargement demonstrated a soft consistency and presented with tenderness and significant bleeding. The associated epithelium was thin, fragile and sloughed off with minor abrasion (Figure 1).

**Figure 2:**
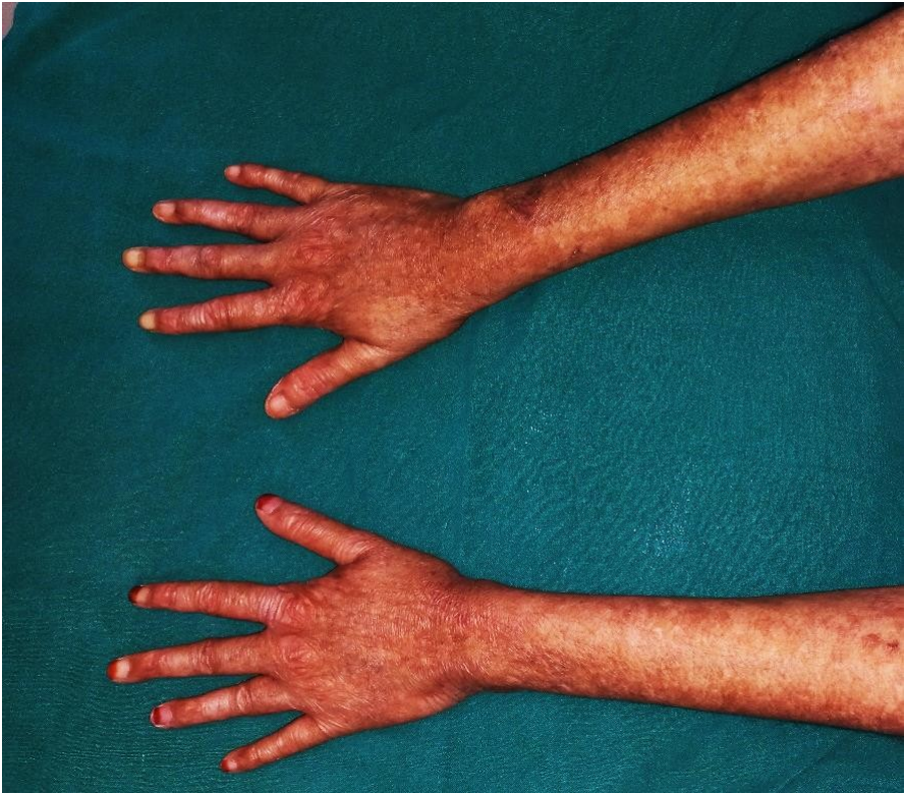
Dermal manifestations of Kindlers syndrome. Generalized skin atrophy coupled with hyper and hypopigmented areas as seen on the dorsal surface of both hands.

We noticed scaly, dry, hyperpigmented, and hypopigmented macules on the dorsal surface of her hands and neck, which led us to others distributed over the entire body. Significant dermal and nail bed atrophy was also noted (Figure 2). On inquiry, the parents reported a history of the patient experiencing acral blistering, primarily affecting the arms, and photosensitivity beginning in infancy, but no therapy was sought.

Upon further examination, the patient was noted to have missing anterior teeth attributed to periodontitis, without any visible caries. There was generalized debris and localized calculus, along with generalized pockets exceeding 5 mm depth. The mobility of teeth ranged from Millers Grade II to Grade III throughout. The gingiva appeared swollen, enlarged, and erythematous, characterized by rolled and cratered margins that visibly separated from the tooth surface upon gentle air stimulation. Additionally, epithelial sloughing was observed even with mild pressure applied.

A panoramic radiograph revealed severe alveolar bone loss with evidence of remnants of bone and scalloping of the cortical plate at the lesion-affected area. Generalized alveolar bone loss and interradicular bone loss were also observed. Multiple missing, impacted, and decayed teeth are also present. The roots of the posterior mandibular teeth appeared relatively short, and bone loss was observed to extend into the apical third, resulting in inadequate bone support and a floating tooth appearance (Supplementary Figure S1).

## 3 Management

The patient/parents were unwilling for any further mucosal or skin biopsies, and hence the diagnosis was essentially clinical. The parents additionally told us that their child may have a syndrome in their reminiscence, but they were unable to provide us with enough details to diagnose her condition. The patient's prior hospital records were also unavailable. For a conclusive diagnosis, the patient was referred to the dermatology department. Upon evaluating the patient's symptoms, the results of the physical examination, histology, and the concordance with Fischer's Criteria, the dermatologist identified the condition as Kindler syndrome (6).

The patient was taken up for further management after obtaining Informed Consent from the patient's parents. The lesion was excised following surgical aseptic techniques and sterile field recommendations under general anesthesia (Supplementary Figure S2). The resection extended down to the periosteum and was followed by extraction of the associated teeth to eradicate any further foci of infection. The excised tissue sample was sent for histopathologic examination in a 10% formalin solution. A periodontal pack was placed, and the patient was recalled after one week for pack removal and a check-up.

The histopathologic examination revealed mucosa-covered tissue with thinning and ulceration of the epithelium. Stroma showed dense mixed inflammatory infiltrate composed of lymphocytes, plasma cells, neutrophils, and numerous proliferating blood vessels and foreign body multinucleated giant cells. The histopathological characteristics are consistent with a clinical diagnosis of Pyogenic Granuloma (Supplementary Figure S3).

An endocrinology opinion was also sought given the high General Random Blood Sugar (GRBS) values and from the ophthalmology department to rule out the possibility of diabetic retinopathy. About 5 days after hospital admission, her clinical course improved, and she achieved adequate glycaemic control. Her insulin infusion was titrated to achieve target blood glucose levels of 100–170 mg/dl.

The dermatologist also advised Medicinal liquid paraffin, (LIQUIN-LIQUID), and an emollient containing Propylene Glycol and Diazolidinyl Urea (Emolene cream) for topical application. Parents were educated about sun safety measures such as the necessity of using sunscreen with a sun protection factor (SPF) of at least 30, dressing the child in long-sleeved dresses, and limiting their child's time spent outdoors. Topical antibiotic ointment was also prescribed to be used in case of infected blisters and a periodic visit to the dermatology department for clinical evaluation of suggestive lesions, if any.

Moreover, children with Kindler syndrome require nourishment not only to support regular growth but also to replenish nutrients lost through open wounds, facilitate rapid wound healing, and enhance the body's ability to combat infections in areas of damaged skin. Hence, the patient was assigned to a dietitian for nutritional support to minimize nutritional deficits, maximize growth, and encourage pubertal development and sexual maturation. The nutritional requirements were determined based on the THINC score and the Clinical Practice Guidelines for Nutrition Support in EB babies and children. As the patient was anemic, hemoglobin levels were checked, and an oral iron supplement was administered to address iron deficiency.

Ten days following the last appointment, the patient was recalled for evaluation. All the surgical locations showed satisfactory healing. A full mouth Scaling and root planing with instruction for home care and regular maintenance cleanings were advised for improvement in the condition of the gingiva and her oral comfort.

**Figure 3:**
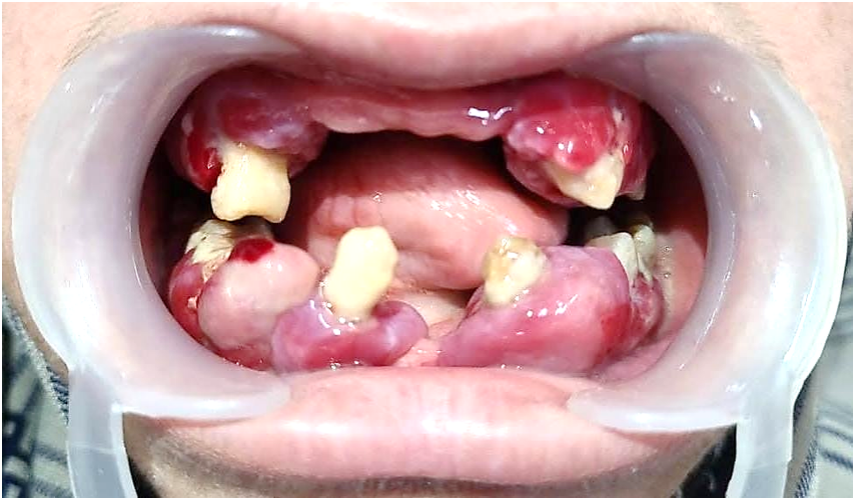
Clinical picture after 1-month follow-up. The remaining soft tissue overgrowths were indicated for excision together with the extraction of the remaining natural teeth owing to the poor periodontal status and Miller's Grade III mobility.

**Figure 4:**
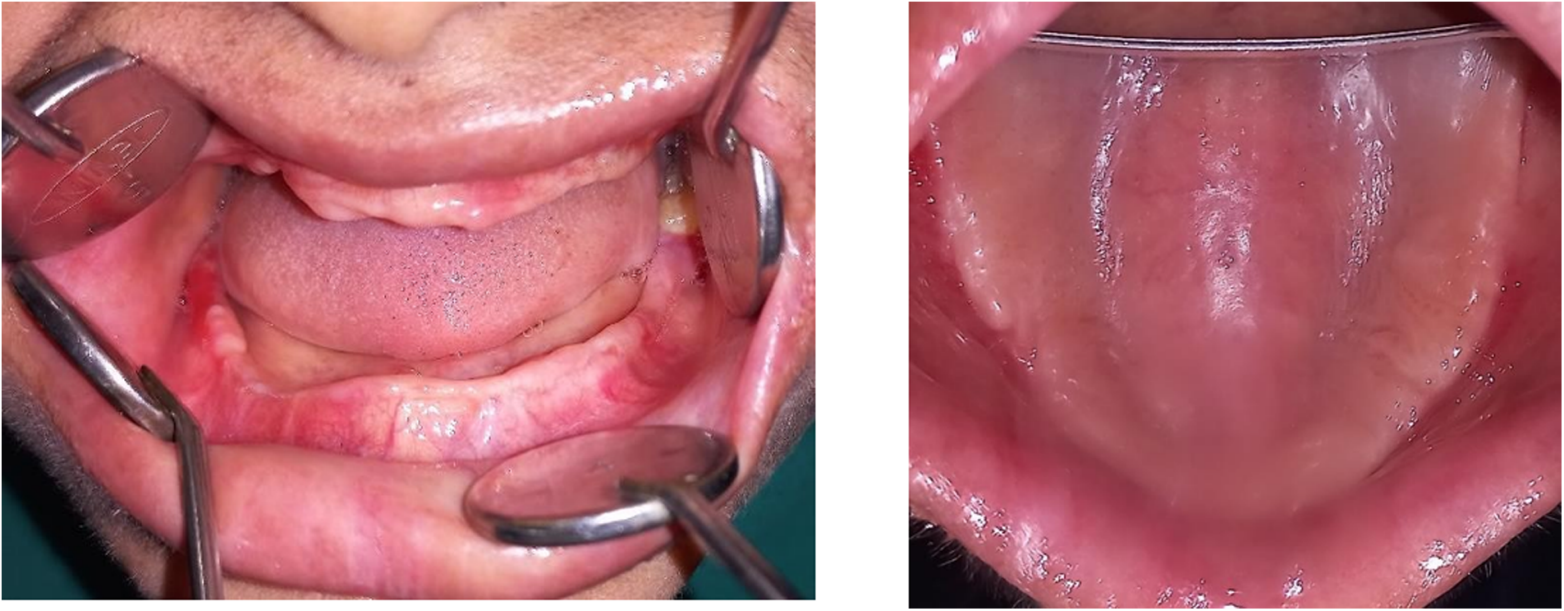
Clinical picture post-two-month follow-up showing edentulous maxillary and mandibular ridges.

However, the patient failed to maintain regular follow-up appointments resulting in a worsening of her periodontal status. There was soft tissue cratering, with spontaneous bleeding and increased mobility on all teeth (Figure 3). Extraction of related teeth is rarely essential, but all the remaining teeth except 38 were indicated for extraction due to pockets extending to more than 5 mm and bone loss extending to the apical region in this case. Her HbA1c declined significantly to 7.1, with blood sugars falling within the optimal range. On a three-month follow-up, all the surgical sites showed satisfactory healing, but with a painful ulceration of the alveolar ridge mucosa (Figure 4).

Due to the patient's history of poor oral hygiene maintenance and the uncertainty in predicting soft tissue responses around implants in patients with KS, the feasibility of successful implant rehabilitation is currently in question. Therefore, the initial treatment plan involves the delivery of provisional complete dentures to restore occlusion, achieve prompt aesthetic improvement, and allow the patient to acclimate to removable prosthetics before proceeding to definitive treatment. The parents and patient were educated about the importance of regular follow-up visits, proper brushing techniques, and effective plaque control measures.

Once we establish consistent adherence to these protocols by the patient and parents, the next step will be scheduling for implant-retained overdentures. This phase will be followed by regular clinical assessments every 2–3 months and annual radiographic evaluations to monitor and prevent peri-implantitis.

## 4 Discussion

While previous studies have documented periodontal complications associated with KS, this case underscores the potential for a particularly severe clinical course when co-existent with DM. This rare confluence of factors—KS, co-morbid DM, and family history suggestive of consanguinity—culminated in a fulminant presentation of an aggressive pyogenic granuloma and severe advanced periodontitis at an exceptionally young age.

Structural defects of epithelium and connective tissue, especially those associated with abnormalities in the basement membrane can act as risk factors at the initiation and progression of severe early-onset periodontal destruction. In this case, the aggressive nature of the oral lesions can be attributed to the synergy between KS and Diabetes Mellitus.

While Kindlin-1 deficiency primarily disrupts integrin function in the oral epithelium, its effects extend beyond cell adhesion. KS can cause mutations in β-1 Integrin-associated adhesions, mainly in the skin and mucous membranes leading to epithelial fragility (7). This compromised epithelial integrity likely plays a significant role in the increased susceptibility to infections and periodontal breakdown observed in KS patients.

Furthermore, research by Maier et al. suggests that KS triggers the upregulation of pro-inflammatory cytokines, including Interleukin-1 beta (IL-1β), Interleukin-6 (IL-6), and Tumour Necrosis Factor alpha (TNF-α), within the skin and keratinocytes (8). This chronic inflammatory state can further contribute to tissue destruction and periodontal disease progression.

Epidemiological research has conclusively shown Type 1 diabetes mellitus (T1DM) as a significant risk factor for periodontitis (9). Earlier clinical studies have demonstrated that children under 18 years old with T1DM exhibited increased attachment loss and bone loss compared to controls, despite similar plaque scores (10). The biological connection between periodontitis and T1DM during childhood and adolescence can be explored at various levels, including the shared inflammatory pathways, transient bacteremia arising from periodontal infections, and systemic immune responses to chronic peripheral infections (11).

Inflammation stands as a well-recognized hallmark in the pathogenesis of both T1DM and periodontitis. Notably, studies have revealed elevated levels of Prostaglandin E2 (PGE2), TNF-α, and IL-1β and functional impairment of polymorphonuclear leukocytes (PMNs) in T1DM individuals, presenting with gingivitis or periodontitis (12, 13).

While pyogenic granuloma (PG) is a relatively common benign soft-tissue tumor of the oral cavity, its clinical presentation in this case deviated from the norm. Typically, PG appears as an erythematous and hemorrhagic, elevated papule or nodule, most frequently located in the anterior maxilla (14) Notably, alveolar bone involvement is rarely associated with PG (15). However, in this case, the radiographic examination revealed significant resorption of the alveolar bone at the site of the lesion.

We hypothesize that the increased vascularity and large size of the lesion, in this case, were induced by the exacerbated release of various endogenous and angiogenic factors in response to the increased fragility of the oral mucosa due to KS and impaired wound healing and ischemia, caused by uncontrolled high blood glucose levels. Contributing to the pathophysiology of Kindlers, in this case, would be the diabetic skin challenges due to the build-up of AGEs, their interaction with RAGEs that trigger diverse intracellular signaling cascades, and altered keratinocyte activities due to insulin's effect on keratinocyte proliferation, differentiation, and migration.

In the absence of a definitive diagnostic test, the diagnosis of KS in this case was primarily clinical, based on the patient's symptoms, medical and family history, and the exclusion of other possible dermatological and oral manifestations of systemic conditions.

Langerhans cell histiocytosis was postulated as a possible diagnosis based on the patient's age, cutaneous and oral exam findings, and the fact that she has not yet entered puberty. The patient's increased gingival volume, mass in her maxillary anterior region, copious gingival bleeding, mobile teeth, loss of supporting alveolar bone akin to advanced periodontal disease, and floating tooth appearance in the 47,37 region were all suggestive of LHC (16).

The differential diagnosis also considers other poikilodermatous syndromes like Bloom syndrome, Rothmund-Thomson syndrome, dyskeratosis congenita, and Cockayne syndrome. Although both KS and Bloom syndrome exhibit poikiloderma, Bloom syndrome presents with additional characteristic features not seen in KS, such as telangiectasia (dilated blood vessels), severe growth restriction, and a significantly increased risk for malignancies at a young age (17). Rothmund-Thomson syndrome shares poikiloderma and photosensitivity with KS. However, unlike KS, Rothmund-Thomson syndrome is also characterized by sparse hair, hypogonadism (impaired development of sex organs), and cataracts (17, 18). Patients with Cockayne syndrome develop a photo-distributed rash with erythema, atrophy, and hyperpigmentation. Additionally, they exhibit dwarfism, progressive vision problems, hearing loss, and microcephaly—features absent in KS (19). In patients with dyskeratosis congenita, there is a male preponderance with mental retardation, and a triad of reticulated hyperpigmentation, nail dystrophy, and leukoplakia (20).

While Kindler syndrome is a rare genetic disorder with limited treatment options, studies and case reports have demonstrated the significant benefits of non-surgical periodontal therapy and regular dental maintenance for preserving periodontal health in these patients (4). As illustrated in this case, maintaining regular dental recall appointments is essential in Kindler syndrome to prevent accelerated periodontal breakdown. The neglect from caregivers and inconsistent dental care likely contributed to the deterioration of the periodontium and the extent of gingival overgrowth observed at presentation.

Therefore, as dental professionals, prompt identification and timely intervention are crucial for the comprehensive dental management of KS. It is essential to investigate for any associated pathological conditions or systemic illnesses that can accelerate periodontal breakdown or cause abnormal oral manifestations, as observed in this case.

Once a diagnosis of KS is suspected, a multidisciplinary team approach is essential. Collaboration with dermatologists, pediatricians, periodontists, prosthodontists, implantologists, and maxillofacial surgeons can ensure a comprehensive evaluation and the initiation of appropriate treatment protocols tailored to the specific needs of the patient.

Suggested recommendations for the dentist managing a patient with KS:

General recommendations:

1. Development of customized oral hygiene measures- Advice an extra soft toothbrush, demonstrate gentle and effective brushing techniques, fluoridated toothpaste and regular salt water rinses.
2. Diet counselling- Advise a diet rich in energy-providing foods and emphasize intake of micronutrients and vitamins, particularly Vitamin C and Zinc, to aid in wound healing and immune modulation.
3. Consider Metronidazole + Chlorhexidine gel application/Chlorhexidine-Hyaluronic acid mouthwash regimen for two weeks every two months, along with regular saltwater gargling.
4. Educate on reducing sugary intake in terms of frequency and amount to minimize oral health risks.
5. Monitor BMI at every recall interval to track nutritional status.
6. Schedule recall appointments every 3 months initially or shorter, as needed, to ensure adherence to the oral hygiene management protocol.
7. Prescribe topical anesthetic medications for blisters as necessary to manage discomfort.
8. Implement strategies to address the psychological impact of oral complications on the patient's quality of life such as positive reinforcement.

In-office procedures:

1. Lubricate the lips and all instruments with Vaseline to reduce drying and adhesion.
2. Apply topical anesthetic gel and consider local anesthesia to minimize discomfort.
3. Use suction tips, mirrors, or cheek retractors on a cotton roll to avoid direct contact with the mucosa and reduce trauma-induced blistering.
4. Re-evaluate individual tooth prognosis and consider extraction of hopeless teeth.
5. Use the lowest power of ultrasonic tips for subgingival calculus removal. Use a soft toothbrush or gauze dipped in Chlorhexidine for debris removal.
6. Perform scaling and polishing with abundant irrigation for thorough cleaning and avoid use of three- way syringe.
7. Utilize lasers when feasible for a more comfortable and minimally invasive approach to manage gingival enlargements, pocket formation, and caries removal.
8. Follow oral prophylaxis with polishing and fluoride application to strengthen tooth enamel.
9. Restore carious teeth with GIC and protective crown placement where indicated to maintain oral health.
10. Evaluate and reinforce oral hygiene maintenance techniques during each visit.

## 5 Conclusion

Wiery-Kindler syndrome (WKS) is a rare genodermatosis characterized by increased fragility and blistering of the skin and mucous membranes. It is also associated with accelerated periodontal breakdown and other oral manifestations. Despite limited curative treatments, regular maintenance with procedures like scaling and root planing could significantly enhance prognosis. Dental professionals are encouraged to recognize the benefits of supportive periodontal therapy and to educate and motivate patients about the positive impacts of dental interventions on overall health outcomes.

## Data Availability

The raw data supporting the conclusions of this article will be made available by the authors, without undue reservation.
