# Supplementary material for: Unusual oral manifestation of Kindler syndrome: a case report and review of literature
Source: Front Oral Health. 2024 Sep 5;5:1430698. doi: 10.3389/froh.2024.1430698 (PMC11410757; doi:10.3389/froh.2024.1430698)
Supplement: Supplementary file 1 [file Datasheet1.docx]

Supplementary Material

Unusual Oral Manifestation of Kindlers Syndrome: A case report and review of Literature

Rahul Bhandary^1^†, Geethu Venugopalan^1*^†, Padmaraj Hegde^1^†

† Equal contribution and first authorship: These authors contributed equally to this work and share first authorship

Department of Periodontology, A B Shetty Memorial Institute of Dental Sciences, Nitte (Deemed to be University), Deralakatte, Karnataka, India

*** Correspondence:**

Corresponding Author: Geethu Venugopalan
drgeethuv@gmail.com

Supplementary Data


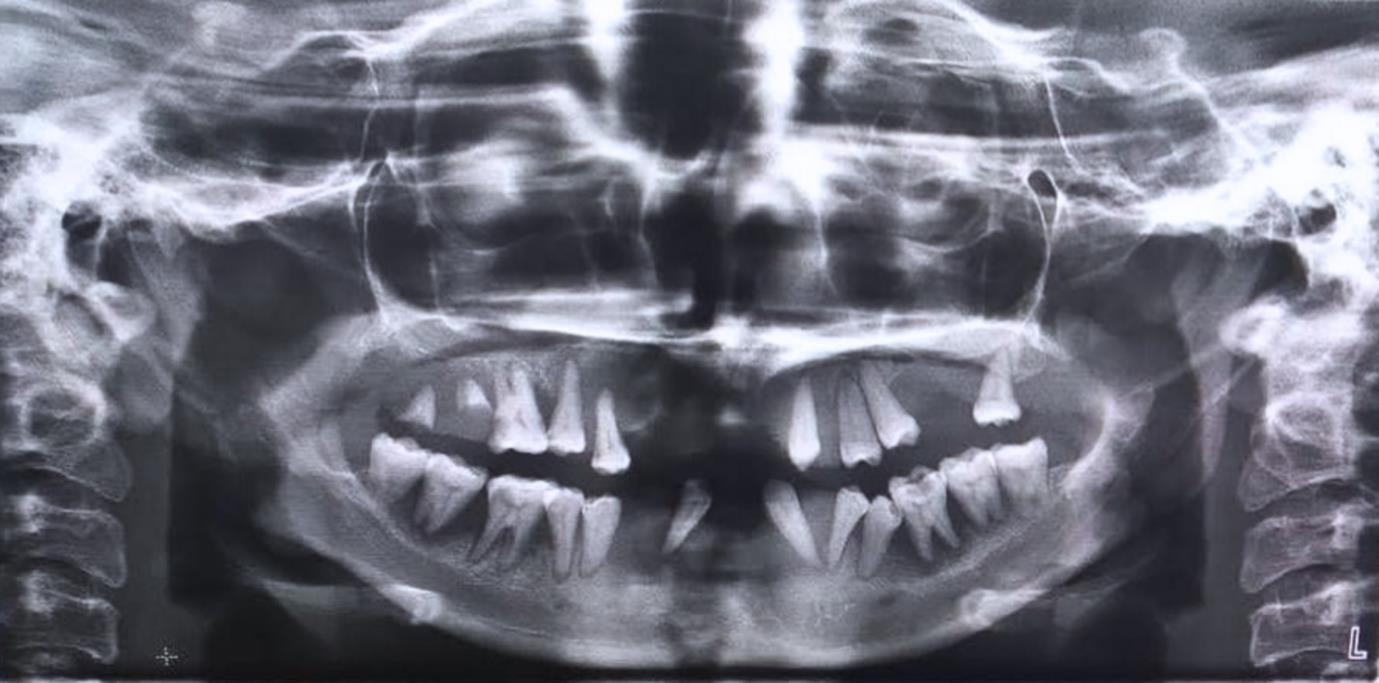


**Supplementary Figure 1.** Panoramic radiograph 10 days following the surgery showing a severe alveolar bone loss in the lesion-affected area with evidence of remnants of bone and scalloping of the cortical plate. Generalized alveolar bone loss and inter radicular bone loss can also be observed. Multiple missing, impacted, and decayed teeth are also seen. Inadequate bone support and a "floating tooth" appearance can be observed in the posterior mandibular teeth due to short roots and bone loss that extends into the apical third.


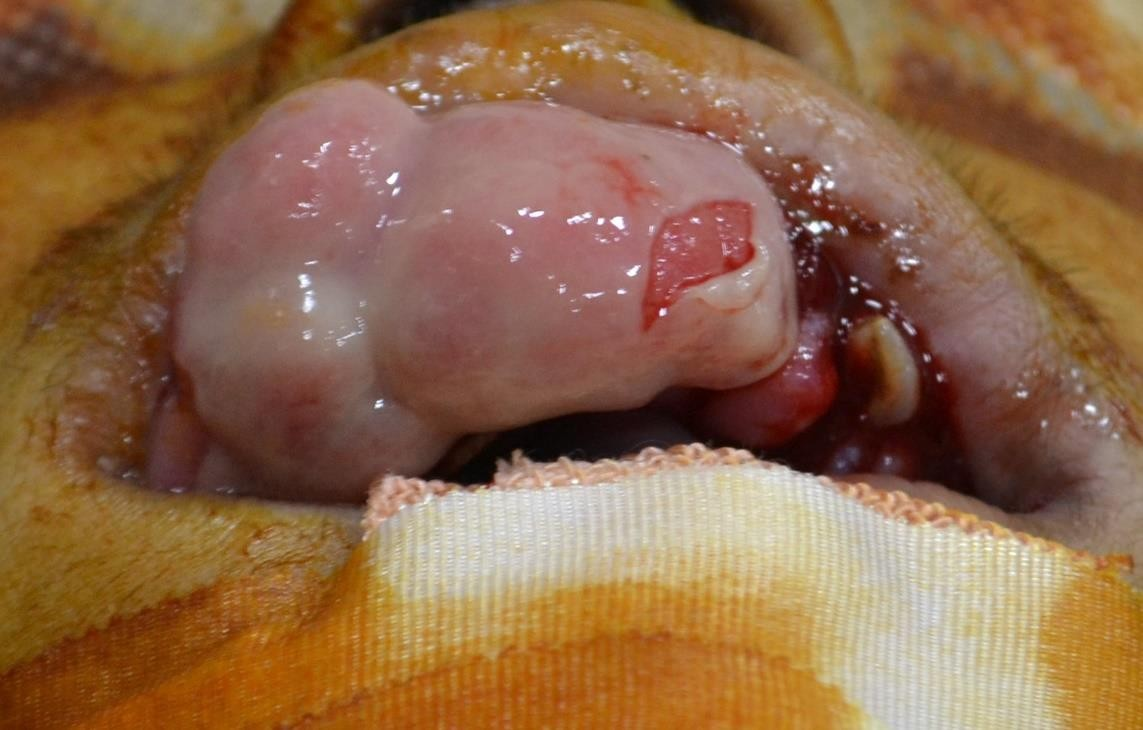
S**upplementary Figure 1.** Massive pyogenic granuloma in the maxillary anterior region showing erythematous, friable skin, gingival bleeding, and the associated teeth.


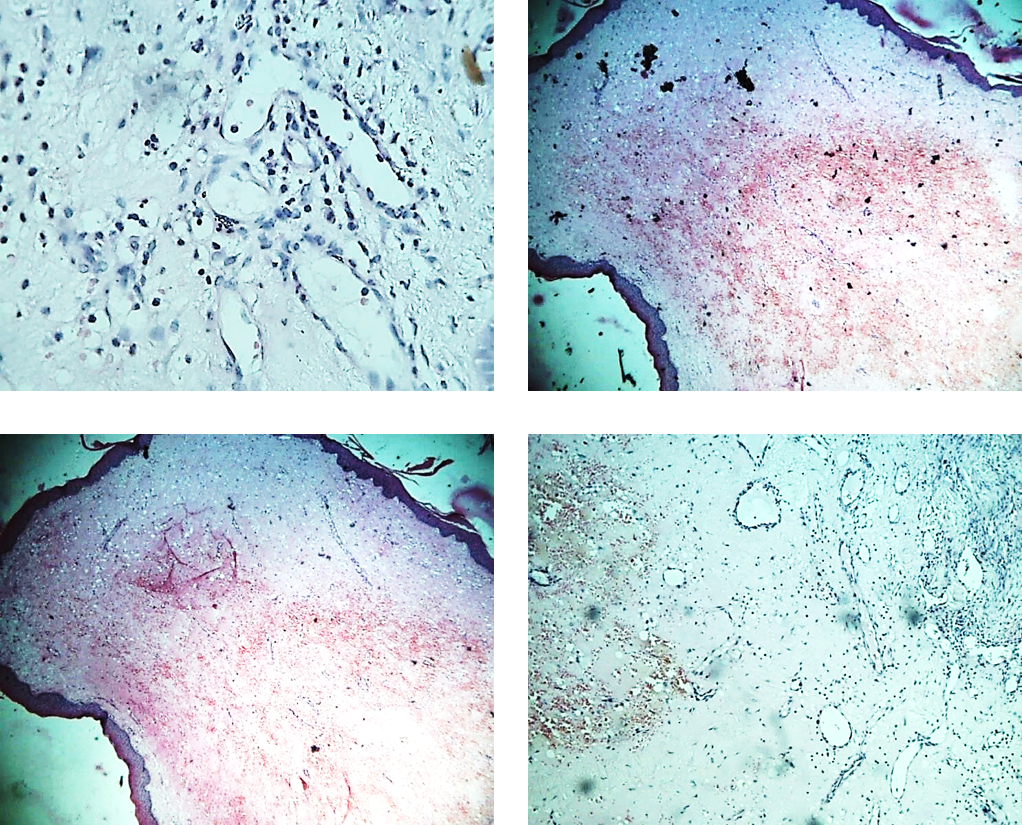


**Supplementary Figure 3.** Histopathological examination of the H&E stained sections shows epithelium and underlying connective tissue. The epithelium is the stratified squamous para-keratinized epithelium and shows hyperkeratotic areas and a few areas with ulceration. The underlying connective tissue shows fibrovascular reactive tissue containing abundant young proliferating blood capillaries filled with red blood cells, numerous plump active fibroblasts, and densely infiltrated acute and chronic inflammatory cells.
